# Isolation and Screening of Polyhydroxyalkanoates Producing Bacteria from Pulp, Paper, and Cardboard Industry Wastes

**DOI:** 10.1155/2013/752821

**Published:** 2013-10-29

**Authors:** Anish Kumari Bhuwal, Gulab Singh, Neeraj Kumar Aggarwal, Varsha Goyal, Anita Yadav

**Affiliations:** ^1^Department of Microbiology, Kurukshetra University, Kurukshetra, Haryana 136119, India; ^2^Department of Biotechnology, Kurukshetra University, Kurukshetra 136119, India

## Abstract

*Background*. Polyhydroxyalkanoates (PHAs) are storage materials that accumulate by various bacteria as energy and carbon reserve materials. They are biodegradable, environmentally friendly, and also biocompatible bioplastics. Unlike petrochemical-based plastics that take several decades to fully degrade, PHAs can be completely degraded within a year by variety of microorganisms into CO_2_ and water. In the present study, we aim to utilize pulp, paper, and cardboard industry sludge and waste water for the isolation and screening of polyhydroxyalkanoates (PHAs) accumulating bacteria and production of cost-effective PHB using cardboard industry waste water. *Results*. A total of 42 isolates showed black-blue coloration when stained with Sudan black B, a preliminary screening agent for lipophilic compounds, and a total of 15 isolates showed positive result with Nile blue A staining, a more specific dye for PHA granules. The isolates NAP11 and NAC1 showed maximum PHA production 79.27% and 77.63% with polymer concentration of 5.236 g/L and 4.042 g/L with cardboard industry waste water. Both of the selected isolates, NAP11 and NAC1, were classified up to genus level by studying their morphological and biochemical characteristics and were found to be *Enterococcus sp., Brevundimonas sp.* and, respectively. *Conclusion*. The isolates *Enterococcus sp*. NAP11 and *Brevundimonas sp*. NAC1 can be considered as good candidates for industrial production of PHB from cardboard industry waste water. We are reporting for the first time the use of cardboard industry waste water as a cultivation medium for the PHB production.

## 1. Introduction

Plastic materials that have been universally used in our daily lives are now causing serious environmental problems. Millions of tons of these nondegradable plastics accumulate in the environment per year. For efficient management of used-plastic materials, recycling is one solution. Another solution to reduce plastic residue is the use of biodegradable plastics [1, 2] and among them polyhydroxyalkanoic acids (PHAs) are drawing much attention. Polyhydroxyalkanoic acids (PHAs) are common intracellular compounds found in bacteria, archaea, and in few eukaryotes such as yeasts and fungi. PHAs are carbon and energy reserve polymers produced in some microorganisms when carbon source is in plentiful and other nutrients such as nitrogen, phosphorus, oxygen or sulfur are limited. PHAs are found to accumulate in varieties of microorganisms as reserve food material for example, *Alcaligenes latus, Ralstonia eutropha, Azotobacter beijerinckii, Bacillus megaterium*, and *Pseudomonas oleovorans*, including some fungi and archaea. Among the members of PHA family, polyhydroxybutyrate (PHB) is the most common biodegradable polymer and promising alternative to synthetic nondegradable plastics. These polymers are accumulated intracellular membrane enclosed inclusion up to 90% of the cell dry weight under conditions of nutrient stress and act as energy reserve material. It has similar mechanical properties as those of the oil-derived conventional plastics like polypropylene or polyethylene which can be molded, made into films, spun into monofilaments, and used to make heteropolymers with other synthetic polymers and many more applications in agriculture, packaging, and medical field being biodegradable and also immunologically compatible with human tissue [3]. Recently, another application of PHAs is reported as biofuel [4].

In spite of these interesting properties, industrial production of PHAs is still not well established. In the 1950s, North-American Company W.R. Grace Co. made the first attempt to produce PHB at commercial level. However, this process was not successful due to low production efficiency and a lack of suitable purification method. Then, in the 1970s, Imperial Chemical Industries (ICI, UK) started producing PHAs by using a mutant stain *Cupriavidus necator*, NCIB 11599 from various carbon sources such as 1,4-butanediol, 1,6-hexanediol, and butyrolactone. The commercial product was recognized as Biopol. The patents were later sold to Zeneca, then to Monsanto and are currently the property of Metabolix, Inc. (USA). Commercially, some other biopolyester products with different monomer composition are also produced with trade names such as poly(3HB-co-3HV) Nodax, poly(3-hydroxybutyrate-co-3-hydroxyalkanoate) poly(3HB-co-3HA) as Biogreen, and poly(3HB) as Biomer [5]. But the large scale production was halted at commercial level due to the high production cost as compared with the oil-derived plastics [1, 6]. With the aim of commercializing PHA, great efforts have been employed to reduce the production cost by the development of bacterial strains and more efficient fermentation/recovery process [7, 8]. From the literature, it has been found that the major cost in this biopolymer production is the cost of the substrate [9, 10] which accounts for more than 50% of production cost [5, 11] and makes the difference in price of poly-3-hydroxybutyrate (P3HB) from Biomer about 12 times the cost of polypropylene [12]. To solve this problem, inexpensive substrate, renewable substrates, waste material, and waste water are used as nutrient source for microorganisms for PHA production. Various types of waste products have been used for PHB production because it provides dual benefits of utilizing the waste and cost-effective production of biodegradable microbial bioplastic.

Cardboard industry is one of the parts of pulp, paper, and packaging industry. Cardboard industry includes two main industries corrugated cardboard industry and noncorrugated cardboard or paper-board industry. Waste water from chemical and mechanical pulping contains 12–25 kg of BOD/t of ADP, but the BOD discharges are 3 to 10 times higher in chemimechanical pulping as compared to mechanical pulping. Nitrogen and phosphorus are also present in waste waters and released from the pulping process of raw material such as wood, agricultural waste, and paper waste. Waste waters released from pulp and paperboard mills are typically rich in carbohydrates but poor in fixed nitrogen. If we consider the scenario of India, pulp and paperboard industry around 905.8 million m^3^ of water is consumed and around 695.7 million m^3^ of waste water is discharged annually. The largest part of the fresh water is used in sheet formation on the cardboard machine (200 m^3 ^h^−1^) and the smallest quantity is used in stock preparation (90 m^3 ^h^−1^ for thickening). Furthermore, the treatment of waste stream to purified effluent needs much effort and is very difficult, because the waste stream often contains various organic compounds. So instead of costly treatment, we can exploit the waste water directly for cultivation of PHB accumulating microorganisms. In this study, polyhydroxyalkanoic acids (PHAs) accumulating bacterial strains were isolated and screened using cardboard industry waste water as a sole carbon source with dual benefit of utilizing the waste and cost-effective production of biodegradable microbial bioplastic.

## 2. Material and Methods

### 2.1. Isolation of Polyhydroxyalkanoic Acids (PHAs) Producing Bacteria

 For the isolation of PHA producing bacteriam activated sludge and waste water were collected from pulp, kraft, and cardboard manufacturing industry from Khanna pulp and paper mills at Amritsar and Topara Kurdh, Yamuna Nagar, India, respectively. The samples were stored at room temperature until analysis. In 99 mL sterilized water, 1 gm of sludge sample was dissolved. Then, the sample was serially diluted in sterile distilled water and followed by plating on the nutrient agar medium with 1% glucose. For isolation from waste water sample, 1 mL of water sample was added in 99 mL sterilized water. After serial dilution (10^−3^ to 10^−7^), 1 mL of each dilution was spread on carbon rich nutrient agar plate. For the rapid detection and isolation of PHB producing bacteria, 0.02% alcoholic solution of Sudan black B was applied to stain bacterial colonies and the plates were kept undisturbed for 30 min. The excess dye was then decanted and plates were rinsed gently by adding 100% ethanol. Colonies unable to incorporate the Sudan black B appeared white, while PHB producers appeared bluish black [13].

### 2.2. Screening for PHA Producing Bacteria

Sudan black B positive isolates were checked for PHA production by Nile blue A staining, a more specific stain for Polyhydroxyalkanoic acids (PHAs) by a more rapid and sensitive, viable colony method [14]. This dye at concentrations of only 0.5 *μ*g/mL was directly included in carbon rich nutrient agar medium (glucose 1%, beef extract 0.3%, peptone 0.5%, sodium chloride 0.8%, and agar 1.5%) and growth of the cells occurred in the presence of the dye. This allowed an estimation of the presence of PHAs in viable colonies at any time during the growth experiment and a powerful discrimination between PHA-negative and PHA-positive strains. The PHA accumulating colonies, after Nile blue A staining, showed bright orange fluorescence on irradiation with UV light and their fluorescence intensity increased with the increase in PHA content of the bacterial cells. The isolates which showed bright orange fluorescence on irradiation with UV light after Nile blue A staining were selected as PHA accumulators. 

### 2.3. Pretreatment of Cardboard Industry Waste Water

Untreated cardboard industry effluent was collected from the cardboard industry, Topara Kurdh, Yamuna Nagar, Haryana, India, and stored at 4°C until used for analysis. The effluent was first filtered through the muslin cloth and then by rough filter paper to remove the undesired suspended solid materials from waste water. After this pretreatment step, cardboard industry waste water was used as quantification and production medium for PHA production by selected bacteria.

### 2.4. Extraction and Quantitative Analysis of PHA

The PHB production was observed in 250 mL Erlenmeyer flask containing 50 mL of treated cardboard industry waste water, as production medium under stationary conditions of growth. After 72 h of incubation at 37°C, culture broth was centrifuged at 8000 rpm for 15 min. The pellet along with 10 mL sodium hypochlorite was incubated at 50°C for 1 h for lyses of cells. The cell extract obtained was centrifuged at 12000 rpm for 30 min and then washed sequentially with distilled water, acetone, and absolute ethanol. After washing, the pellet was dissolved in 10 mL chloroform (AR grade) and incubated overnight at 50°C and was evaporated at room temperature [15]. After evaporation, 10 mL of sulphuric acid was added to it and placed in water bath for 10 min at 100°C. This converts polyhydroxyalkanoic acids (PHAs) into crotonic acid, which gives maximum absorbance at 235 nm [16, 17]. PHB (Sigma Aldrich) was used as standard for making standard curve. For quantitative analysis of PHA, cell culture was grown as described earlier and cell pellet was dried to estimate the dry cell weight (DCW) in units of g/L [18]. Residual biomass was estimated as the difference between dry cell weight and dry weight of extracted PHA [19]. This was calculated to determine the cellular weight and accumulation other than PHAs. The percentage of intracellular PHA accumulation is estimated as the percentage composition of PHA present in the dry cell weight:

(1) Residual biomass (g/L) =DCW (g/L)−Dry weight of extracted PHA (g/L),PHA accumulation (%)  =Dry weight of extracted PHA (g/L)      ×100%/DCW (g/L).

### 2.5. Morphological Characterization and Biochemical Identification of PHA Producing Bacteria

Microscope Stereo Olympus was used to observe the morphology of bacterial colonies grown on nutrient agar. The growth characteristics such as structure, shape, color, margin, surface characteristics, surface upwards, smell, elevation, opacity, end of cells, cell's arrangement, and Gram-staining of the bacterial colonies were observed to characterize the bacterial colonies.

Various biochemical tests were performed in selected PHB producing bacteria, namely, indole production test, methyl red and Voges-Proskauer, citrate utilization test, and H_2_S production for their biochemical characterization. The fermentative utilization of various carbohydrates was also followed for 48 hrs at 37°C by inoculating the isolates separately in the defined medium to which various sugars like xylose, mannose, maltose, sucrose, raffinose, dextrose, trehalose, fructose, glucose, ribose, lactose, rhamnose, esculin, inulin, mannitol, arabinose, sorbitol, and melibiose were added. 

### 2.6. Polymer Analysis 

#### 2.6.1. ^1^H-NMR Spectroscopy and Thermal Gravimetric Analysis (TGA)

The identity of individual monomer unit was confirmed by proton nuclear magnetic resonance (^1^H-NMR) spectroscopy. ^1^H-NMR spectra were acquired by dissolving the polymer in deuterochloroform (CDCl_3_) at a concentration of 10 mg/mL and analyzed on a Bruker Avance II 500 spectrometer at 22°C with 7.4 ms pulse width (30° pulse angle), 1 s pulse repetition, 10,330 Hz spectral width, and 65,536 data points. Tetramethylsilane was used as an internal shift standard. Thermal gravimetric analysis (TGA) was performed using a TGA instrument (Mettler-Toledo, TGA/SDTA 851, USA) calibrated with indium. The temperature was ramped at a heating rate of 10°C/min under nitrogen to a temperature (700°C) well above the degradation temperature of the polymers.

### 2.7. FT-IR Analysis

FT-IR analysis of the polymer sample was carried out on MB-3000, ABB FTIR spectrophotometer in the range 4000–600 cm^−1^. 

### 2.8. GC-MS Analysis

Purified polymer, prepared as described before, was dissolved in 2 mL of chloroform and then 2 mL of methanol was added and acidified with 3% (v/v) H_2_SO_4_ and heated at 100°C for 3.5 h for depolymerization and methanolysis of polyesters and 3 *μ*L was injected into GCMS-QP 2010 Plus model. The samples were injected in the splitless mode and the injection temperature was 260°C and column oven temperature was 100°C.

## 3. Result and Discussion

### 3.1. Isolation and Selection of PHA Producing Bacteria

**Figure 1:**
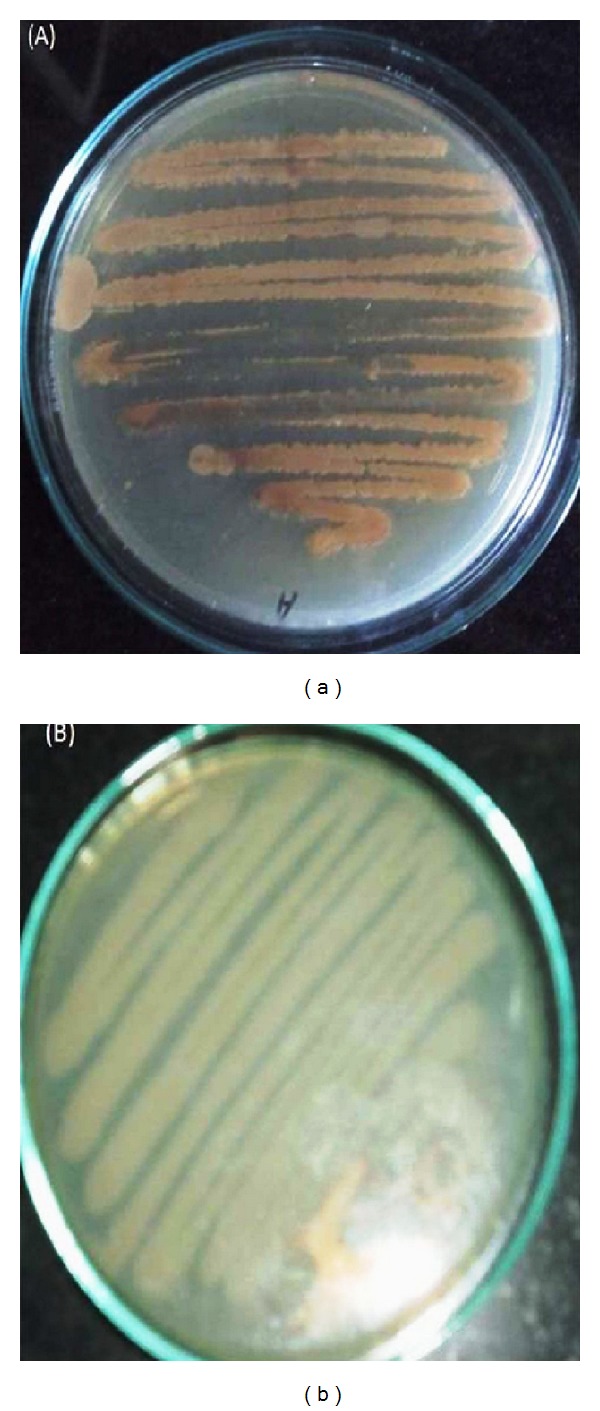
(a) Pink/orange florescence under UV light by PHB producer isolate NAP11. (b) No florescence under UV light by Non-PHB producer with Nile blue A staining by viable colony method.

A wide variety of bacteria are known to accumulate PHA. Today, approximately 150 different hydroxyalkanoic acids are known to be incorporated into polyhydroxyalkanoates [20], with microbial species from over 90 genera being reported to accumulate these polyesters [21]. These bacteria have been reported from various environments, but only a few from the waste water and sludge ecosystems. For the rapid detection and isolation of PHB producing bacteria, 0.02% alcoholic solution of Sudan black B and Nile blue A staining viable colony method [14] was used. The isolation of PHA producing bacteria was done from cardboard manufacturing industry waste water and pulp cardboard and kraft industry sludge. A large proportion about 35% of isolated bacteria produced PHA as energy reserve material. A total of 42 isolates showed black-blue coloration when stained with Sudan black B, a preliminary screening agent for lipophilic compounds, and a total of 15 isolates showed positive result with Nile blue A staining (Figure 1), a specific dye for the of PHA granules. Both gram-positive and gram-negative bacteria showed PHA production, but gram-positive bacteria dominated the waste material microflora of pulp, kraft, and cardboard manufacturing industry. Teeka et al. [22] used this method to screen the potential PHA producing bacteria from soil, and Ramachandran and Abdullah [23] also observed the colonies formed on nutrient rich medium under ultraviolet light (UV) to screen for the pink fluorescence which indicated the presence of PHA producers. Kitamura and Doi [24] first demonstrated this viable colony method on agar plates; they induced the isolates to accumulate PHA by culturing in E_2_ medium containing 2% (w/v) glucose before Nile blue A staining. The PHA accumulating colonies, after Nile blue A staining, showed bright orange fluorescence on irradiation with UV-light and their fluorescence intensity increased with increase in PHA content of the bacterial cells.

### 3.2. Production, Extraction, and Estimation of PHA with Cardboard Industry Waste Water as a Sole Carbon Source

The PHA-positive isolates selected after Nile blue A staining were grown in 50 mL cardboard industry waste water in Erlenmeyer flasks and were employed to extract PHA after 2 days of incubation under stationary conditions of growth. The PHA from the isolates was extracted by the hypochlorite and chloroform method [15] as described earlier. The isolates NAP11 and NAC1 showed maximum PHA production 79.27% and 77.63% (Table 1) with cardboard industry waste water and were selected for further biochemical identification and chemical characterization. 

Organic matter from wastes and waste waters has high BOD and COD values, and hence microorganisms can grow, utilizing the nutrient present in waste water, and can convert them into valuable compounds and polymers. Based on this idea, many researchers reported the PHA production from various industrial waste materials. PHA production by *A. vinelandii* from swine waste liquor was studied by [25]. The raw liquor supported the production of only 0.43 g/L PHA, at a polymer content of 37% w/w, whereas twofold dilution and supplementation with 30 g glucose/L allowed a PHA concentration of 5.5 g/L at a 58.3% w/w polymer content. Few researchers have proposed coupling PHA production to biological waste water treatment [26–28]. Ceyhan and Ozdemir [29] reported polyhydroxybutyrate (PHB) production from domestic waste water using *Enterobacter aerogenes* 12Bi strain with good yield ranging from 16.66 to 96.25% (w/w). The use of pure *C. necator* cultures to produce PHAs from waste waters has been explored by Ganzeveld et al. [30]. They used a supernatant, obtained by centrifuging fermented organic waste, as the sole carbon source for the production of P(3HBco-3HV), and obtained a maximum polymer concentration of 1.13 g/L at a polymer content of 40.8% in 45 h. Cardboard industry waste water is typically rich in carbohydrates but poor in fixed nitrogen, due to the high C/N ratio. This high carbon-nitrogen ratio favors the growth of PHA producing bacteria. It is the first time that cardboard industry waste water is used for the isolation, screening, and production of polyhydroxyalkanoates. This waste has high BOD and COD values 680–1250 mg/L and 3400–5780 mg/L and COD/BOD ratio between 3.9 and 5 [31], which is suitable for microbial growth.

**Figure 2:**
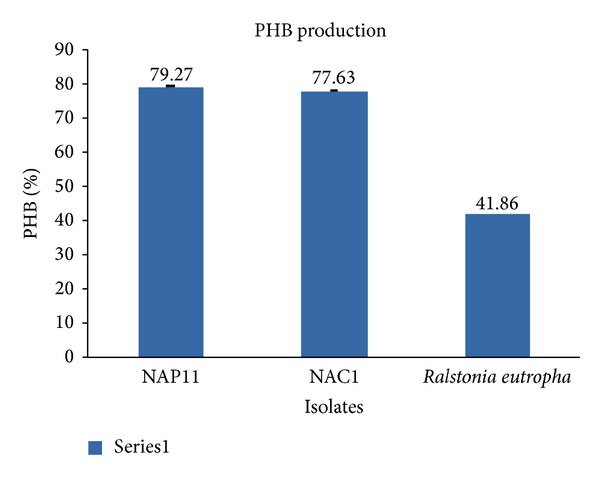
Comparison of PHB production from selected isolates with *Ralstonia eutropha. *

Extracted PHA of selected isolates was quantified and its efficiencies were compared with the standard. Standard pure culture of *Ralstonia eutrophus MTCC no. 1473* was used for PHA production with cardboard waste water producing a polymer concentration of 2.974 g/L and PHB content up to 41.30% with cardboard industry waste water. The selected isolates NAP11 from pulp sludge have produced 79.27% w/w PHA with polymer concentration of 5.236 g/L using cardboard waste water which are 37% higher as compared to standard stain of *Ralstonia eutrophus *(Figure 2). The other NAC1 isolates showed PHA production up to 77.63% with polymer concentration of 4.042 g/L under stationary conditions of growth.

### 3.3. Morphological and Biochemical Characterization of Selected Isolates

By using Bergey's Manual of Determinative Bacteriology [32] and by ABIS Online-Advanced Bacterial Identification Software, bothisolates were classified up to genus level using the morphological and biochemical characteristics (Table 2). NAP11 and NAC1 were found to be *Enterococcus sp*., and *Brevundimonas sp*., *respectively*. Other researchers also reported these genuses from the waste effluents. Silva et al. [33] studied the ecology of *Enterococci *and related bacteria in raw and treated waste water from a treatment plant receiving domestic and pretreated industrial effluents. The predominant species found in the raw waste water were *Enterococcus hirae, Enterococcus faecium, and Enterococcus faecalis.* Jiang et al. [34] isolated 3,851 in total *Enterococci* isolates from eight individual source categories including feces from animals and birds, soil, and sewage water samples to establish antibiotic resistance analysis (ARA). Reddy and Mohan [35] also reported the *Enterococcus italicus* sp. in mixed consortia in waste water treatment and produced PHA up to 71.4%. During their study of influence of substrate load and nutrient concentration (nitrogen and phosphorous) on PHA production using waste water as substrate and mixed culture as biocatalyst, they found that PHA accumulation was high at higher substrate load (40.3% of dry cell weight (DCW)), low nitrogen (45.1% DCW), and low phosphorous (54.2% DCW) conditions by mixed consortia containg in *Enterococcus sp*. this paper confirms that Rani et al. [36] reported *Brevundimonas* with other bacteria as the dominant cultured bacteria in microbial diversity in functional pesticide effluent treatment plants (ETPs). *Brevundimonas aveniformis sp.nov*. A stalked species, was isolated from activated sludge by Ryu et al. [37]. *Brevundimonas sp. MIFC* and *Brevundimonas diminuta *was isolated from refinery active sludge and olive mill waste water, respectively, [38] and a *Brevundimonas sp*. were isolated from tannery waste treatment plant [39]. PHA production also reported up to 64% from the acid hydrolyzed saw dust (hydrolyzed wood) by *Brevundimonas vesicularis*. They also optimized the C : N ratio for PHA production in *Brevundimonas vesicularis sp*. and found that C : N proportion of 100 : 3.5 yielded maximum PHA up to 64% of cell dry weight. Thus, they concluded that acid hydrolyzed saw dust can be used as substrate by *Brevundimonas vesicularis sp.* for PHA production [40]. 

### 3.4. Polymer Analysis by ^1^H-NMR Spectroscopy

**Figure 3:**
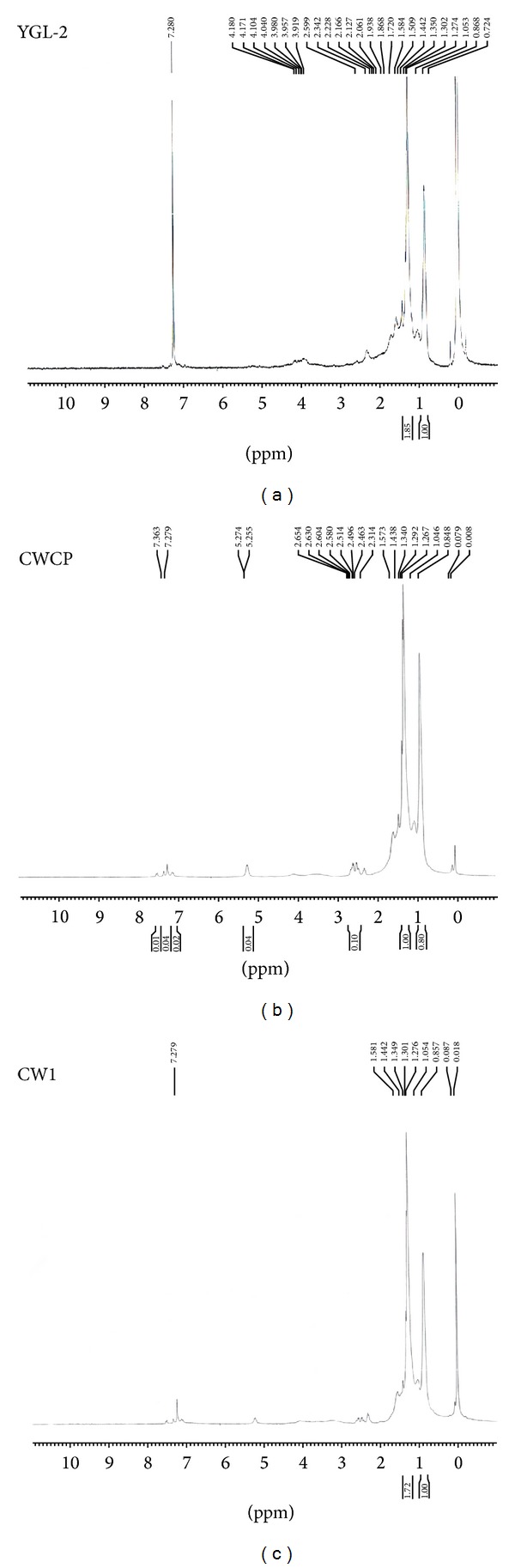
^1^H NMR spectra of extracted PHB from isolates: (a) PHB standard (PHB Sigma Aldrich), (b) NAP11, and (c) NAC1.

 Based on the characterization of the PHA produced by NAP11 and NAC1 through NMR comparison with the standard PHB (Sigma), it was observed that the PHA obtained from NAP11 and NAC1 is having properties similar to that of the standard PHB (Sigma) (Figure 3(a)), so the PHA produced by both bacteria is polyhydroxybutyrate (PHB). The structures of polyesters were investigated by _1_H NMR. The _1_H NMR spectra of the PHAs extracted from *Enterococcus sp*.* NAP11* show the following resonance signals: HC=CH bond at 5.25 ppm, CH_2_O–COOH bond at 2.580 ppm, a high signal at 1.26 ppm that belongs to the hydrogen of methylene in the saturated lateral chain, and a terminal –CH3 group at 0.8 ppm; the _1_H NMR spectra (Figure 3(b)) of the PHAs extracted from *Brevundimonas sp. NAC1* (Figure 3(c)) show the following resonance signals: HC=CH bond at 5.30 ppm, CH_2_O–COOH bond at 2.574 ppm, a high signal at 1.30 ppm that belongs to the hydrogen of methylene in the saturated lateral chain, and a terminal –CH3 group at 0.857 ppm [15]. The ^1^H NMR spectra of the samples and the standard are almost identical, conferring that extracted intracellular compounds are polyhydroxybutyrates (PHBs).

### 3.5. Fourier Transform Infrared Spectroscopy (FTIR)

**Figure 4:**
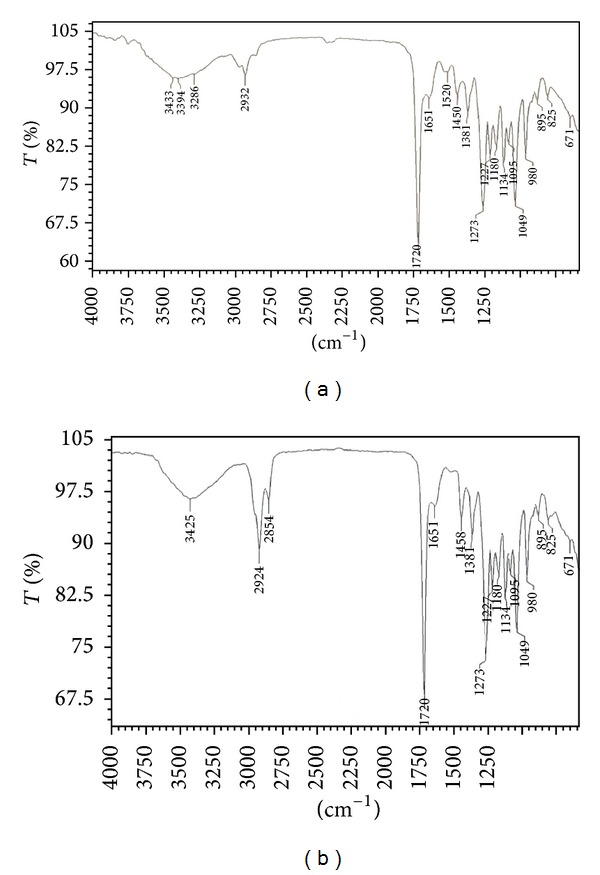
FTIR graph of extracted polymer of (a) NAP11 and (b) NAC1 isolates.

Polymer extracted from NAP11 and NAC1 was used for recording IR spectra in the range 4000–600 cm^−1^. IR spectra (Figure 4) showed two intense absorption bands at 1720 and 1281 cm^−1^ of NAP11 and at 1720 and 1273 of NAC1 specific for C=O and C–O stretching vibrations, respectively. The absorption bands at 2932 and 2954 cm^−1^ are due to C–H stretching vibrations of methyl, methylene groups. These prominent absorption bands confirm the structure of poly-*β*-hydroxybutyrate.

### 3.6. Thermogravimetric Analysis (TGA)

TGA results of NAM5 showed that the *T*_*m*_ is 171.33°C and the enthalpy of PHA fusion is 85.56 J/g. The result showed similarity with the data obtained from standard PHB (176.29°C and 86.49 J/g) [41] and from other studies from the literature also [42, 43].

### 3.7. GC-MS Analysis of Extracted PHA

**Figure 5:**
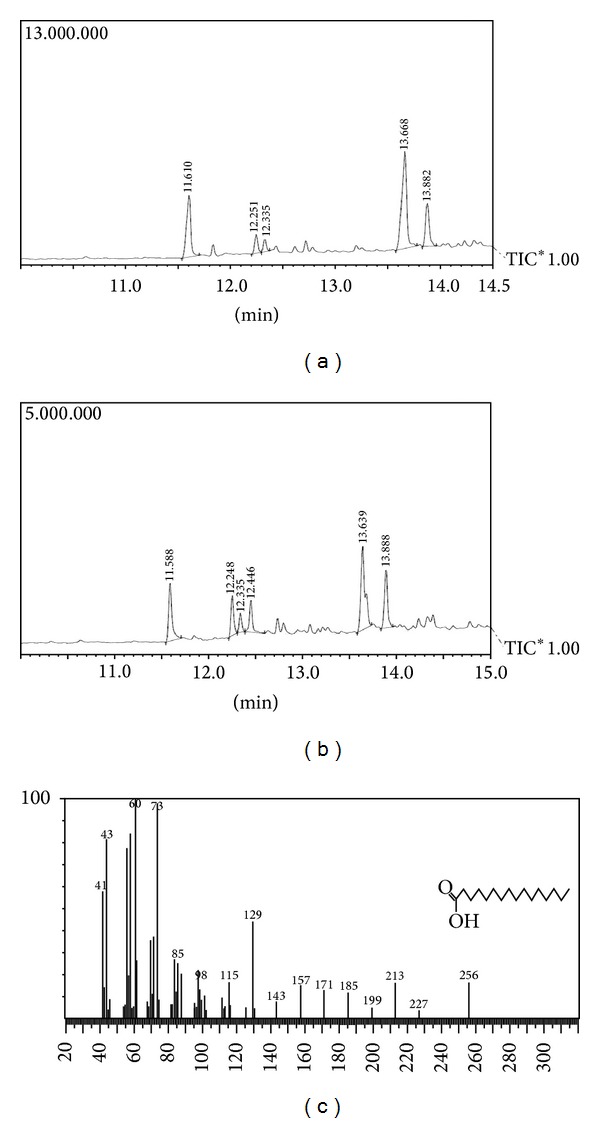
GC/MS analysis of poly(3HB) purified from NAP11 and NAC1 isolates. (a) GC of NAP11 showing main peak at 13.667 min of 3HB dimer. (b) GC of NAC1 showing main peak at 13.667 min of 3HB dimer and 3 other peaks indicating the presence of 3HV in the purified polymer. (c) MS graph showing that the major peak at *m/z* 73 is of hexadecanoic acid.

In this study, the PHB was methanolysed in the presence of sulphuric acid and methanol, and the methanolysed 3HB was then analyzed by GC-MS. Figures 5(a) and 5(b) showed that a common molecular fragment of the 3HB methyl ester ion chromatogram of the PHB was produced. A predominant peak corresponding to the dimer 3HB methyl ester was noted at 13.63 to 13.667 min, respectively, in GC purified product from NAP11 and NAC1, while 3 other major peaks were observed at 11.5, 12.2, and 12.3 min. The retention times and ion fragment patterns of the peaks at 11.6 and 12.2 min were identical to those of the dimer methyl esters of 3HV and 3HBV, respectively, but in very low percentage up to 5% in isolate NAP11 and 20% and 11% in NAC1 isolate, respectively. From the data obtained by GC-MS, the molecular weightof PHB obtained from isolate NAP11 is 256 kDa and from isolate NAC1 is 242 kDa.

## 4. Conclusion

In this study, inexpensive cardboard industry waste water was tried as a carbon source to produce PHA. Different bacterial strains were isolated from cardboard industry waste water and pulp, kraft, and cardboard manufacturing industry sludge and screened for polyhydroxyalkanoate production using cardboard manufacturing industry waste water as a carbon source. The bacterial isolates NAP11 and NAC1 can be regarded as potential strains for the conversion of cardboard industry waste water into PHB. Both of the selected isolates utilized cardboard industry waste water as sole carbon source for growth and PHB biosynthesis, accumulating PHB up to 79.27% and 77.63% of the cell dry mass, respectively. As a conclusion, isolates NAP11 and NAC1 can be considered as good candidates for industrial production of PHB from cardboard industry waste water. Based on the morphological and biochemical characterization, NAP11 and NAC1 were identified up to genus level as *Enterococcus sp*. and *Brevundimonas sp.*, respectively. Currently, these bacterial strains are further investigated to increase the productivity of PHB by the optimization of the process parameters and making the whole process more cost-effective.

## Figures and Tables

**Table 1:** List of PHA accumulating bacteria with source of isolation.

Name of isolate	Source of isolate	Gram reaction	PHA concentration (g/L)	PHA content (%)
NAP11	Pulp industry sludge	+ve	5.236	79.27 ± 0.3
NAP4	Pulp industry sludge	+ve	3.682	65.75 ± 0.2
NAW2	Waste water from cardboard manufacturing industry	−ve	4.012	76.26 ± 0.26
NAW24	Waste water from cardboard manufacturing industry	+ve	4.018	62.90625 ± 0.11
NAW19	Waste water from cardboard manufacturing industry	+ve	3.824	59.52 ± 0.34
NAW23	Waste water from cardboard manufacturing industry	+ve	3.63	58.54839 ± 0.27
NAW27	Waste water from cardboard manufacturing industry	−ve	2.966	51.13793 ± 0.16
NAW34	Waste water from cardboard manufacturing industry	+ve	1.61	40.25 ± 0.18
NAC1	Waste sludge from cardboard manufacturing industry	−ve	4.042	77.63 ± 0.3
NAC24	Waste sludge from cardboard manufacturing industry	+ve	4.006	65.75 ± 0.12
NAC10	Waste sludge from cardboard manufacturing industry	−ve	3.97	64.80 ± 0.16
NAC9	Waste sludge from cardboard manufacturing industry	+ve	3.802	70.92 ± 0.13
NAC12	Waste sludge from cardboard manufacturing industry	+ve	3.682	68.28 ± 0.04
NAK8	Kraft industry sludge	+ve	3.83	71.53571 ± 0.05
NAK31	Kraft industry sludge	+ve	3.366	62.33 ± 0.13
NAK17	Kraft industry sludge	+ve	3.184	63.68 ± 0.18
*Ralstonia eutropha MTCC no. 1473 *	MTCC	−ve	2.974	41.86 ± 0.1

**Table 2:** Morphological and biochemical characters of selected isolates.

Morphological characters	NAP11	NAC1
Colony color	White	Cream
Colony texture	Smooth	Smooth-elevated
Gram reaction	+ve	−ve
Cell shape	Cocci-shaped	Rod-shaped
Cell arrangement	Chain	Chain
Spore formation	−ve	−ve

Biochemical tests		

Citrate utilization test	−ve	−ve
Indole test	+ve	+ve
Methyl red test	−ve	−ve
V-P test	−ve	−ve
H_2_S production test	+ve	+ve
Amylase production test	−ve	−ve
Sucrose	+ve	−ve
Dextrose	+ve	+ve
Mannitol	+ve	+ve
Trehalose	+ve	+ve
D-Arabinose	−ve	−ve
Maltose	+ve	+ve
Glucose	+ve	+ve
Raffinose	+ve	+ve with gas production
Xylose	−ve	−ve
Sorbitol	−ve	−ve
Lactose	−ve	−ve
Mannose	−ve	−ve
Fructose	+ve	−ve
Ribose	−ve	−ve
Inulin	−ve	−ve
Esculin	−ve	−ve
Mellibiose	−ve	−ve
Ramanose	−ve	−ve
